# Fe‐Doped Carbon Dots as NIR‐II Fluorescence Probe for In Vivo Gastric Imaging and pH Detection

**DOI:** 10.1002/advs.202206271

**Published:** 2023-01-03

**Authors:** Qiaoqiao Ci, Yuanyuan Wang, Ben Wu, Emerson Coy, Jiao jiao Li, Daoyong Jiang, Pengfei Zhang, Guocheng Wang

**Affiliations:** ^1^ Research Center for Human Tissues and Organs Degeneration Shenzhen Institute of Advanced Technology Chinese Academy of Science Shenzhen Guangdong 518055 China; ^2^ Guangdong Key Laboratory of Nanomedicine CAS‐HK Joint Lab of Biomaterials Shenzhen Engineering Laboratory of Nanomedicine and Nanoformulations Institute of Biomedicine and Biotechnology Shenzhen Institute of Advanced Technology Chinese Academy of Science Shenzhen Guangdong 518055 China; ^3^ NanoBioMedical Centre Adam Mickiewicz University Wszechnicy Piastowskiej 3 Poznan 61–614 Poland; ^4^ School of Biomedical Engineering Faculty of Engineering and IT University of Technology Sydney Ultimo NSW 2007 Australia

**Keywords:** bioimaging, carbon dots, fluorescence imaging, gastric pH sensing, near‐infrared

## Abstract

Carbon dots (CDs) with excellent cytocompatibility, tunable optical properties, and simple synthesis routes are highly desirable for use in optical bioimaging. However, the majority of existing CDs are triggered by ultraviolet/blue light, presenting emissions in the visible/first near‐infrared (NIR‐I) regions, which do not allow deep tissue penetration. Emerging research into CDs with NIR‐II emission in the red region has generated limited designs with poor quantum yield, restricting their in vivo imaging applications due to low penetration depth. Developing novel CDs with NIR‐II emissions and high quantum yield has significant and far‐reaching applications in bioimaging and photodynamic therapy. Here, it is developed for the first time Fe‐doped CDs (Fe‐CDs) exhibiting the excellent linear relationship between 900–1200 nm fluorescence‐emission and pH values, and high quantum yield (QY‐1.27%), which can be used as effective probes for in vivo NIR‐II bioimaging. These findings demonstrate reliable imaging accuracy in tissue as deep as 4 mm, reflecting real‐time pH changes comparable to a standard pH electrode. As an important example application, the Fe‐CDs probe can non‐invasively monitor in vivo gastric pH changes during the digestion process in mice, illustrating its potential applications in aiding imaging‐guided diagnosis of gastric diseases or therapeutic delivery.

## Graphical abstract

Fe‐doped CDs (Fe‐CDs) exhibiting strong NIR‐II fluorescence emission in the gastric pH range and an excellent linear relationship between NIR‐II fluorescence‐emission and pH values (2.0–6.0) is developed. They are successfully applied as effective probes for non‐invasive real‐time in vivo NIR‐II bioimaging to monitor gastric pH during food digestion in mice even under the influence of antacid drugs (omeprazole).

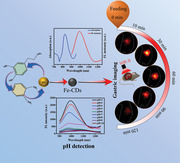

## 1 Introduction

Developing enabling technologies for accurate real‐time bioimaging is the key to improving disease diagnosis. As a prime example, dynamic detection of gastric acidity in vivo is essential for the accurate diagnosis and treatment of gastric diseases and plays an important role in the development of pH‐triggered stomach medicines.^[^ ^1^, ^2^ ^]^ At present, invasive endoscopic examinations are used clinically to assess stomach pH values, which lead to patient discomfort and are not suitable for long‐term monitoring.^[^ ^3^ ^]^ To solve these problems, non‐invasive techniques have been explored to monitor stomach pH, such as ingestible electronic capsules^[^ ^4^ ^]^ and organic dye.^[^ ^5^ ^]^ However, due to the limited tissue penetration depth of the probes used, these techniques still require endoscopy or ex vivo analyses to assist the optical measurements.^[^ ^6^ ^]^ There remains an imminent need to develop a simple, safe, efficient, and accurate non‐invasive bioimaging method for assessing pH, with one of its applications being in vivo gastrointestinal pH detection in physiological and pathological studies.

Fluorescence imaging has become a vital imaging technique due to its excellent spatiotemporal resolution, which can realize real‐time and non‐invasive visualization of biological systems.^[^ ^7^, ^8^, ^9^ ^]^ In comparison with conventional imaging using visible light (400–650 nm) and near‐infrared light in the first bio‐window (NIR‐I, 700–900 nm), imaging using NIR light in the second bio‐window (NIR‐II, 900–1700 nm) has attracted increasing attention due to its distinct advantages of deep penetration capability, high resolution, and a low interfering background since the triggering light has weak interactions with tissues.^[^ ^10^, ^11^ ^]^ To date, a range of organic and inorganic NIR‐II emitters have been applied for in vivo NIR‐II imaging of biological systems, such as organic fluorophores with low molecular weight,^[^ ^12^, ^13^, ^14^ ^]^ conjugated polymers,^[^ ^15^, ^16^ ^]^ carbon nanotubes,^[^ ^17^, ^18^ ^]^ rare earth materials,^[^ ^19^, ^20^ ^]^ quantum dots (QDs),^[^ ^21^, ^22^ ^]^ and others.^[^ ^23^ ^]^ However, organic NIR‐II fluorophores (e.g., cyanine dyes, BODIPY) have been heavily impeded in biomedical applications by the well‐known aggregation‐caused quenching (ACQ) effect.^[^ ^24^ ^]^ On the other hand, existing inorganic NIR‐II fluorophores (e.g., single‐walled carbon nanotubes, rare earth nanoparticles) often have complicated synthesis routes and unknown biological toxicity, hindering their clinical translation.^[^ ^25^ ^]^ There is hence a prime demand both scientifically and clinically to build new NIR‐II fluorescent probes with enhanced overall performance compared to existing options.

Carbon dots (CDs) are known for their excellent cytocompatibility, tunable optical properties (photoluminescence), cost‐effectiveness, simple synthesis routes, and versatility for further functionalization.^[^ ^26^, ^27^, ^28^ ^]^ They have demonstrated great potential for applications in optoelectronic conversion,^[^ ^29^, ^30^ ^]^ bioimaging,^[^ ^31^, ^32^ ^]^ biosensing,^[^ ^33^, ^34^ ^]^ nanomedicine,^[^ ^35^, ^36^ ^]^ and catalysis.^[^ ^37^, ^38^ ^]^ Despite there being a huge range of fluorescent CDs produced in recent years, the vast majority have a short emission wavelength in the blue/green region of the spectrum, with limited penetration depth, and can cause damage to the surrounding tissue. Some CDs with emission wavelengths in the range of deep‐red or NIR‐I (<900 nm) have been recently developed, but most of these are exclusively excited by shorter wavelength light with high energy, which still pose high risks of photodamage to biological tissues.^[^ ^26^ ^]^ Moreover, the majority of existing red‐emissive CDs are insensitive to pH. Carbon dots with NIR‐II emission are currently a rarefied existence. To the best of our knowledge, only two papers reporting NIR‐II CDs are currently available,^[^ ^39^, ^40^ ^]^ and CDs produced in these papers did not show pH responsibility. Li et al.^[^ ^39^ ^]^ produced NIR‐II CDs by a facile hydrothermal method using watermelon as a carbon source. However, the QY of watermelon‐derived CDs was low (≈0.4%), and the fluorescence emission peak is almost at the boundary between the NIR‐I and NIR‐II regions (925 nm). In addition, it has not been found that CDs materials with NIR‐II imaging ability have pH response ability, which liming their biomedical engineering applications. New strategies to produce NIR‐II CDs with higher QY, longer excitation wavelengths, and pH sensing capability will satisfy significant unmet needs for in vivo bioimaging and biosensing.

**Scheme 1:**
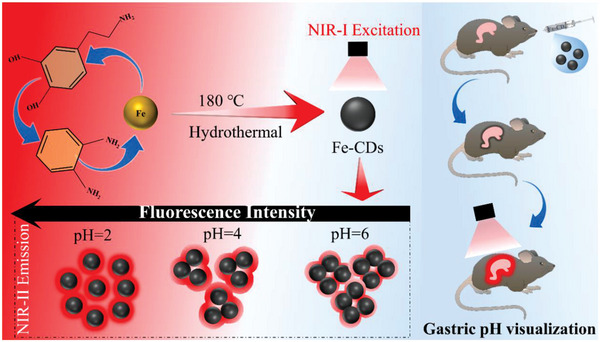
Schematic illustration of novel NIR‐II Fe‐CDs as a nanoprobe for pH biosensing and monitoring of gastric food digestion.

In this work, we for the first time synthesized NIR‐II fluorescent Fe‐doped CDs (Fe‐CDs) with high QY, using dopamine hydrochloride (DA) and o‐phenylenediamine (oPD) as carbon sources and FeCl_3_·6H_2_O as dopant, through a facile one‐pot hydrothermal method. These unique Fe‐CDs showed (1000 nm) at pH 2, with linearly decreasing fluorescence intensity in response to increasing pH in the range of 2 to 6, due to gradually enhanced aggregation of the CDs (**Scheme** **1**). Drawing on these properties in an example application, we successfully demonstrated the capability of Fe‐CDs as a NIR‐II fluorescent probe for in vivo detection and real‐time monitoring of gastric pH changes in a mouse model, during the normal food digestion process, fasting experiments, and treatment with omeprazole drugs.

## 2 Results and Discussion

### 2.1 Synthesis and Characterization of NIR‐II Fe‐CDs

**Figure 1:**
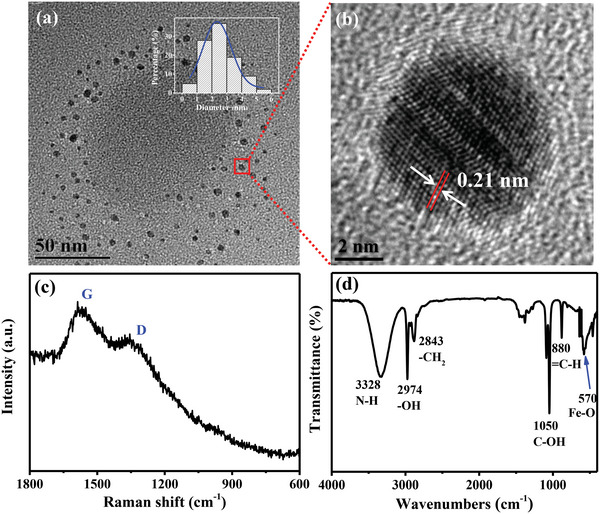
Characterization of Fe‐CDs by a) TEM, b) HR‐TEM, c) Raman spectrum, and d) FTIR.

We prepared NIR‐II Fe‐CDs from DA, oPD, and FeCl_3_•6H_2_O by a one‐pot hydrothermal method. Transmission electron microscopy (TEM) images showed that Fe‐CDs had an average size of 2.35 nm (**Figure** **1**a), and high‐resolution TEM (HR‐TEM) images revealed clear lattice structures with a fringe spacing of 0.21 nm, matching the typical distance between graphite layers (Figure 1b). This indicated graphitization, which was also confirmed through the Raman spectrum by bands at 1578 and 1346 cm^−1^ corresponding to the structure of *sp^2^* (G band) and *sp^3^* (D band) (Figure 1c). It is well‐documented that the extent of graphitization can be determined by the ratio of *sp^3^/sp^2^* carbon.^[^ ^41^ ^]^ In our Fe‐CDs, the ratio of G to D bands (*I*_G_/*I*_D_) was 1.157, indicating a high degree of graphitization. This ratio was also much larger than other existing CDs with NIR‐II emission.^[^ ^39^ ^]^ The dominant graphitic structure of our Fe‐CDs suggests they have excellent NIR‐II emission properties.^[^ ^42^ ^]^

FTIR was used to determine the surface functional groups of Fe‐CDs (Figure 1d). The peaks at 3328 and 2974 cm^−1^ were respectively attributed to the N‐H and ‐OH stretching vibration modes, while the band at 2843 cm^−1^ was attributed to ‐CH_2_. The peaks at 1050 and 880 cm^−1^ corresponded to C—OH and =C—H, respectively.^[^ ^43^, ^44^ ^]^ These results suggested the presence of conjugated aromatic structures together with O‐ and N‐containing groups. Also notable was the peak at 570 cm^−1^ corresponding to catechol‐Fe^3+^ coordination.^[^ ^45^ ^]^ Further validating the intercalation of Fe in the Fe‐CDs, inductively coupled plasma‐mass spectrometry (ICP‐MS) was used, which indicated Fe^3+^ concentration of 0.80% in the Fe‐CDs (Table S1, Supporting Information).

**Figure 2:**
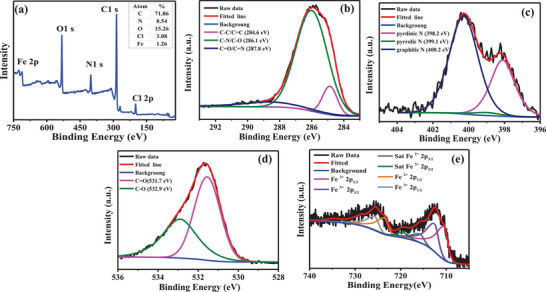
Characterization of Fe‐CDs by a) XPS spectrum, b) C 1s, c) N 1s, d) O 1s, e) Fe 2p3 spectra.

The elemental composition and valence state of Fe‐CDs were identified by X‐ray photoelectron spectroscopy (XPS) spectra (**Figure** **2**a). The Fe‐CDs mainly contained C (71.86 atom%), O (8.54 atom%), N (15.26 atom%), Cl (3.08 atom%), and Fe (1.26 atom%). Three peaks corresponding to C—C/C=C, C—N, and C=O/C=N (284.6, 286.1, and 287.8 eV respectively) were present in the XPS spectrum of C 1s, indicating the existence of heteroatoms in Fe‐CDs (Figure 2b). The N 1s spectrum could be fitted into three peaks (398.2, 399.2, and 400.2 eV), respectively assigned to pyridinic N, pyrrolic N, and graphitic N (Figure 2c). Peaks at 531.7 and 532.5 eV in the O 1s spectrum were respectively assigned to the C=O and C—OH/C—O—C groups (Figure 2d), which was consistent with the C 1s spectrum. Additionally, the XPS spectrum of Fe 2p3 showed different peaks, indicating that the different valence states of Fe included Fe^2+^ and Fe^3+^ (Figure 2e).

Previous studies have shown that C=O and graphitic N play important roles in the red emission of CDs. Specifically, a higher percentage of C=O and graphitic N results in more red shift in the emission fluorescence of CDs.^[^ ^46^ ^]^ In our study, the percentages of C=O and graphitic N calculated from XPS data were 66.44% and 61.85% respectively (Table S2, Supporting Information). The ratio of C=O in Fe‐CDs was much larger than other reported NIR‐II CDs (10.62%),^[^ ^39^ ^]^ pointing to their promising NIR emission properties.^[^ ^47^ ^]^ Interestingly, we also found that CDs synthesized in the absence of Fe^3+^ had significantly lower percentages of C=O (32.23%) and graphitic N (37.71%) compared to Fe‐CDs synthesized under the same experimental conditions (Figure S1, Supporting Information). These findings suggested the important role of Fe^3+^ in the NIR‐II emission of Fe‐CDs, by increasing the proportion of C=O and graphitic‐N.

### 2.2 In Vitro pH Detection

The synthesized Fe‐CDs were used as nanoprobes for in vitro pH detection. In an acidic solution, the absorption peak of Fe‐CDs appeared at 830 nm, with a concurrent emitting fluorescence peak at 1000 nm (Figure S2a, Supporting Information). The optimal fluorescence excitation wavelength (*λ*ex) was 830 nm (Figure S2b, Supporting Information). Using IR‐26 dye as a reference, the QY of Fe‐CDs in water was measured to be 1.27%, much higher than that for the watermelon juice‐derived NIR‐II CDs (QY = 0.4%)^[^ ^39^ ^]^ and suggesting the promising capability of Fe‐CDs as probes for NIR‐II bioimaging.

**Figure 3:**
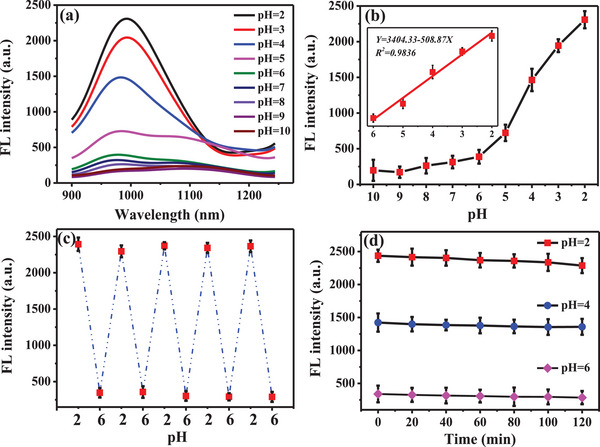
a) Fluorescence emission spectra of Fe‐CDs in solutions with different pH (2.0–10.0). b) Relationship between the fluorescence intensity of Fe‐CDs and pH of the solution. Inset: a linear relationship of fluorescence intensity against pH value is observed over the range of pH 2.0 to 6.0 (*λ*em = 1000 nm). c) Plot showing the reversible switching of Fe‐CDs fluorescence intensity between pH 2.0 and 6.0. d) Fluorescence intensity of Fe‐CDs over a 2 h time period in solutions of pH 2.0, 4.0, and 6.0.

We initially investigated the potential of using Fe‐CDs for in vitro pH sensing in PBS buffer. The fluorescence intensity was strongest at pH 2.0 and gradually weakened with the increase in pH from 2.0 to 10.0 (**Figure** **3**a). There was an excellent linear relation between fluorescence intensity and pH values within the range of 2.0–6.0 (R^2^ = 0.9836, Figure 3b). To confirm that changes in fluorescence intensity of Fe‐CDs were exclusively caused by pH changes, the effects of a range of biologically significant substances (including anions, glutathione, vitamin C, and others) were investigated. The fluorescence signal from Fe‐CDs remained remarkably stable even in the presence of these interfering substances (Figure S3, Supporting Information), speaking to the excellent potential of Fe‐CDs to allow accurate in vivo pH monitoring.

To monitor dynamic pH changes in a biological environment, an ideal pH probe should be able to reversibly measure pH in real‐time. To test the reversibility of the Fe‐CDs in pH sensing, they were placed in PBS buffers with different pH values adjusted using Na_2_HPO_4_ and NaH_2_PO_4_. The fluorescence intensity of Fe‐CDs was shown to be well maintained even after five cycles of pH changes between 2 and 6 (Figure 3c). Furthermore, when placed in solutions with different pH of 2, 4, and 6 over an extended period of time, the fluorescence intensity of Fe‐CDs did not significantly vary within 2 h in all three solutions (Figure 3d). These results suggest that Fe‐CDs have a high degree of fluorescence stability for bioimaging applications.

We also studied the effects of reaction conditions on the fluorescence emission of Fe‐CDs. Fe‐CDs synthesized under different pH conditions showed significant variations in their fluorescence response to changing pH values (Figures S4, S5, Supporting Information). In solutions with pH range of 2 to 10, the fluorescence intensity of Fe‐CDs produced at pH 5.0, 6.0, and 7.0 was relatively stronger than those produced at other pH values. Among these, the Fe‐CD synthesized at pH 5.0 showed the best linear relationship with changes in pH value for those in the range of 2.0–6.0.

To investigate the influence of individual reactants of Fe‐CDs (DA, oPD, and FeCl_3_) on their fluorescence properties, different combinations of reactants were subjected to hydrothermal treatment using the same conditions as those used for Fe‐CDs synthesis. Among all combinations, only the ternary component system consisting of DA, oPD, and FeCl_3_ could produce CDs with NIR absorption.

### 2.3 Response Mechanism of Fe‐CDs to pH

**Figure 4:**
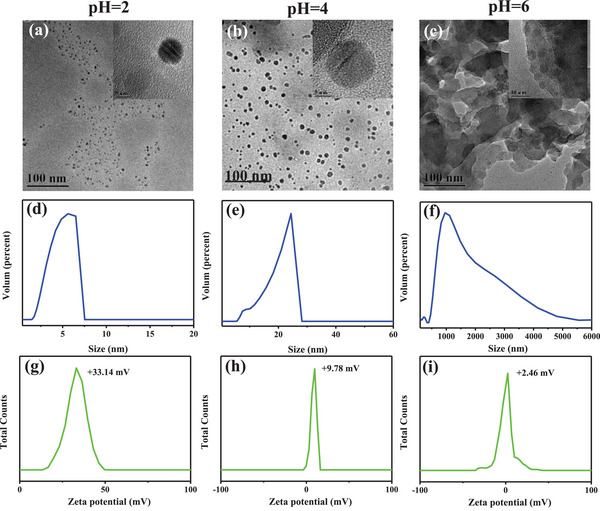
a–c) TEM and HR‐TEM images, d–f) hydrodynamic diameters, and g–i) zeta potential measurements of Fe‐CDs under different pH conditions.

To understand the mechanism by which Fe‐CDs respond to changes in pH, we observed their morphologies by TEM (**Figure** **4**a–c) and measured their hydrodynamic diameters (Figure 4d–f) and zeta potential (Figure 4g–i) under different pH conditions. At pH 2.0, TEM images showed that the Fe‐CDs were monodispersed with an average size of 5 nm (Figure 4a). With the increase in pH to 4.0, Fe‐CDs aggregated and the particle size became bigger with an average of 25 nm (Figure 4b). When the pH increased to 6.0, the Fe‐CDs showed more extensive aggregation, resulting in even larger particle sizes of ≈1 µm (Figure 4c). DLS measurements further confirmed the TEM findings, where the hydrodynamic diameters of Fe‐CDs increased from ≈5 nm to ≈1 µm with increasing pH from 2 to 6 (Figure 4d–f). These phenomena were likely related to pH‐dependent changes in surface zeta potential. At pH 2, the surface potential of Fe‐CDs was highly positive at +33.14 mV (Figure 4g), likely due to the protonation of the surface amine group.^[^ ^48^ ^]^ This high surface potential can oppose particle aggregation by electrostatic repulsion. An increase in pH to 4.0 reduced the surface zeta potential to +9.78 mV (Figure 4h), due to reduced protonation of the amine group leading to moderate aggregation of Fe‐CDs. Further increase to pH 6.0 resulted in further reduction of surface potential to +2.46 mV (Figure 4i) and severe particle aggregation, which almost completely quenched the fluorescence of Fe‐CDs. It is interesting to note that the pH‐induced particle aggregation also led to changes in the color of the Fe‐CDs solution at different pH (Figure S7, Supporting Information). The Fe‐CDs solution at pH 2 exhibited a bright yellow color, which gradually darkened to an ebony color when the pH increased to 6. No obvious color changes were observed with further pH increases from 6 to 9.

### 2.4 In Vitro Cytocompatibility

To evaluate the cytocompatibility of Fe‐CDs, in vitro cell culture experiments using 4T1 cells, RAW 264.7 cells, and 293T cells were performed, and cytotoxicity was measured using a standard Cell Counting Kit‐8 (CCK‐8) assay. As shown in Figure S8 (Supporting Information), Fe‐CDs did not induce obvious cytotoxicity on 4T1, RAW 264.7, and 293T cells even when the concentration reached 200 µg ml^−1^. These results suggest that the Fe‐CDs have good biocompatibility.

### 2.5 NIR‐II Penetration Depth in Ex Vivo Tissue

**Figure 5:**
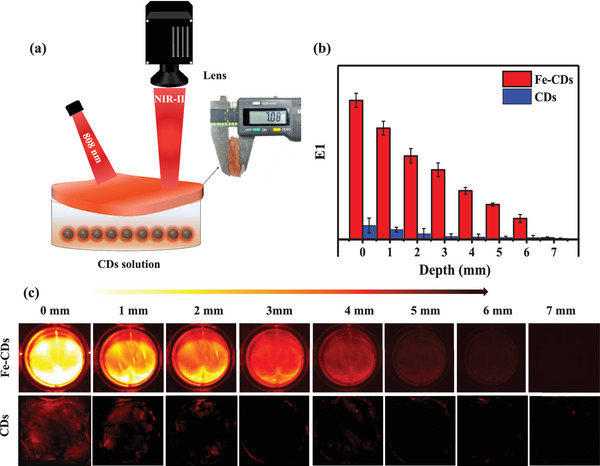
a) Schematic illustration of the set‐up for measuring penetration depth of Fe‐CDs emission (*λ*_ex_ = 808 nm; *λ*_em_ = 1000 nm long pass). The Fe‐CDs solution at pH 2 was covered with porcine tissue of known thickness (0 to 7 mm) in a 24‐well plate. b) Mean emission intensity and c) Fluorescence images of Fe‐CDs and control CDs at different tissue penetration depths.

Prior to in vivo bio‐imaging, we estimated the fluorescence intensity of Fe‐CDs at different penetration depths using ex vivo porcine tissue. Porcine tissue with a known thickness (0 to 7 mm) was used to cover a petri‐dish containing Fe‐CDs in PBS at pH 2.0 (**Figure** **5**a). To measure the penetration capacity of the light emitted by Fe‐CDs, changes in fluorescence intensity when Fe‐CDs were placed under tissues of different thicknesses were detected by charge‐coupled device (CCD) at a wavelength of 1000 nm, and the carbon dots synthesized without Fe ions (CDs) were used as a control. The fluorescence intensity of Fe‐CDs decreased gradually with increasing tissue thickness (Figure 5b,c). The high brightness of fluorescence images acquired using Fe‐CDs for tissue thicknesses ≤4 mm was sufficient for reliable imaging. Remarkably, fluorescence signals from Fe‐CDs were still detectable even beneath 6 mm of tissue. In comparison, the control CDs showed weak fluorescence at all tissue thicknesses, with a very shallow penetration depth leading to almost no visible fluorescence beneath tissues thicker than 1 mm.

Molecular fluorophores are currently widely utilized for NIR‐II imaging, including IR‐26 and ICG (NIR‐I and NIR‐II), etc. However, they are confronted with problems of being prone to quenching and relatively high cytotoxicity. The observed tissue penetration of Fe‐CDs fluorescence in this study was much better than that for IR‐26 and ICG.^[^ ^14^ ^]^ Recently, advanced anti‐quenching molecular fluorophores have been developed by Wang et al for in vivo high‐contrast imaging and pH sensing.^[^ ^14^ ^]^ The tissue depth limit of our Fe‐CDs for reliable imaging with high accuracy was comparable to that demonstrated by this molecular fluorophore. It is also worth noting that the fluorescent intensity of this molecular fluorophore had a linear response over a pH range of 1.0–4.0, while the pH‐responsive range of our Fe‐CDs was higher at 2.0–6.0. Since gastric pH normally varies from 1.0 to 7.0，^[^ ^49^ ^]^ our developed Fe‐CDs are more suitable for the monitoring of gastric pH. In another study, a lanthanide‐based near‐infrared *τ* probe for fluorescence lifetime imaging of upper gastrointestinal pH achieved a maximum in vivo tissue penetration depth of 3 mm.^[^ ^50^ ^]^ Nevertheless, this fluorescent probe remained in the stomach for a relatively short period (less than 2 h) and only detected pH changes within the range of 4 to 7, suggesting limited applications in the long‐term detection of gastrointestinal pH changes or patterns of pH changes in more acidic environments. With these considerations, our Fe‐CDs are a competitive candidate compared to existing technologies for gastrointestinal imaging and pH sensing, due to their capacity to provide high tissue penetration depth, dynamic pH monitoring, long‐term in vivo stability, and high cytocompatibility.

### 2.6 In Vivo pH Imaging and Quantitative Analysis

To validate whether Fe‐CDs can successfully monitor in vivo gastric pH changes in real‐time, two groups of mice were respectively fed with Fe‐CDs solution and Fe‐CDs/Omeprazole (OMG) solution. OMG is a commonly used antacid to neutralize stomach pH, and we expect OMG administration to attenuate the fluorescence signal of Fe‐CDs. Both groups were first imaged non‐invasively, with Fe‐CDs in the stomach beneath 2–4 mm tissue depth, followed by invasive imaging of the dissected stomach with Fe‐CDs in gastric fluid covered by ≈1 mm gastric wall (Figure S9, Supporting Information). For mice in the Fe‐CDs group, non‐invasive imaging showed strong fluorescence at the stomach site (Figure S9a,b, Supporting Information), verifying the high tissue penetration ability of the Fe‐CDs. Exposing the stomach by removing the overlying tissues only slightly enhanced the image brightness (Figure S9c,d, Supporting Information), proving that the Fe‐CDs could provide reliable non‐invasive pH imaging for tissue depths at least up to 4 mm. For mice administered with Fe‐CDs/OMG, no fluorescence signal was detected, which was as expected since OMG induces an increase in gastric pH which would quench the fluorescence from Fe‐CDs (Figure S9e,f, Supporting Information). Although very weak fluorescence signals were detectable during invasive imaging, the brightness of the image was too low for clear visualization (Figure S9g,h, Supporting Information). To prove that the fluorescence signals arose from the stomach rather than other sites, the stomach was dissected and taken out from sacrificed animals for direct imaging (Figure S10, Supporting Information), which verified the non‐invasive and invasive imaging results.

To further verify that the fluorescence signal was from Fe‐CDs rather than other substances in the mouse stomach, the stomachs of mice administered with only Fe‐CDs without OMG were first subjected to NIR fluorescence imaging, and then opened to remove the gastric content and image the gastric inner wall. Strong NIR‐II fluorescence was detected in mouse stomachs fed with Fe‐CDs only (Figure S11a,b, Supporting Information), with even stronger and highly intense fluorescence detected on the inner stomach wall after gastric contents were removed (Figure S11c,d, Supporting Information). These results indicate that Fe‐CDs can be absorbed onto the inner side of the stomach, and generate fluorescence that is reflective of stomach pH while being unaffected by the gastric content.

**Figure 6:**
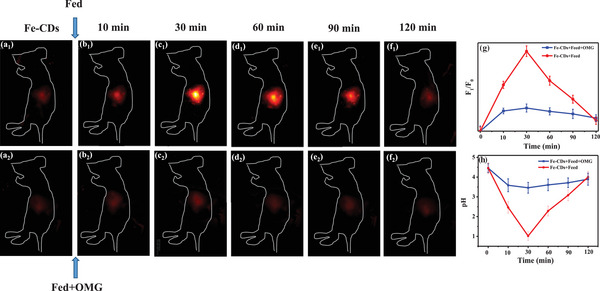
a–f) NIR fluorescence intensity imaging of mice before and after oral administration of Fe‐CDs with or without OMG, and monitored for 120 min following feeding (*λ*ex = 808 nm; *λ*em = 1000 nm long pass). g) Ratio of mean intensity of fluorescence imaging, with h) calculated pH based on fluorescence intensity.

We further investigated the ability of Fe‐CDs in real‐time monitoring of dynamic pH changes in vivo during normal digestion. Two groups of mice were respectively fed with Fe‐CDs and Fe‐CDs/OMG, followed by feeding with food, and their stomachs were monitored for changes in fluorescence intensity over a period of 120 min (**Figure** **6**a–f). The fluorescence signal in the Fe‐CDs group gradually increased since the time of feeding, reaching its peak at 30 min, consistent with the expected time of maximum decrease in gastric pH due to gastric acid production during digestion.^[^ ^49^ ^]^ Over the 2 h period, fluorescence intensity gradually decreased from the peak value, returning to a baseline level similar to that observed before feeding, again corresponding to the expected time course of the digestion process. In the group fed with OMG in addition to Fe‐CDs, the fluorescence signal also peaked followed by a gradual decrease to baseline over the 2 h period, although the peak intensity was significantly lower than in the group not fed with OMG. This was expected since the OMG would have neutralized gastric acid and prevented the drop in gastric pH normally experienced during digestion.

To investigate the linearity of changes in Fe‐CDs fluorescence intensity in response to an in vivo pH gradient, five groups of mice were fed with Fe‐CDs in solutions of different pH (from 2 to 6) and then subjected to NIR imaging (Figure S12a, Supporting Information). The in vivo response of Fe‐CDs was similar to that observed in vitro, where an excellent linear relationship was observed between decreasing fluorescence intensity and increasing pH (Y = 3.464 — 0.553x, R^2^ = 0.988) (Figure S12b, Supporting Information).

From the above findings, we estimated the real‐time, in vivo pH of gastric fluid based on the NIR fluorescence intensity of Fe‐CDs in the mouse stomach during digestion. The starting pH value of gastric fluid was calculated to be 4.2, which decreased to a peak value of 1.2 at 30 min after food intake, and then gradually returned to the original pH value over 2 h (Figure 6h,i). These gastric pH changes are consistent with those expected during normal digestion.^[^ ^5^, ^14^ ^]^ Clinically, it has been difficult to monitor real‐time changes in gastric pH, which is also affected by the presence or absence of food in the stomach. Standard clinical assessment still relies on invasive endoscopic tests or gastric juice extraction, which are invasive and cause significant patient discomfort, and are not suitable for long‐term monitoring. Our Fe‐CDs provide a simple, reliable, and accurate method that is non‐invasive and also cost‐effective for real‐time monitoring of gastric pH, with a linear response range and high tissue penetration due to NIR‐II emission, endowing great potential for future clinical applications.

### 2.7 In Vivo Clearance of Fe‐CDs

**Figure 7:**
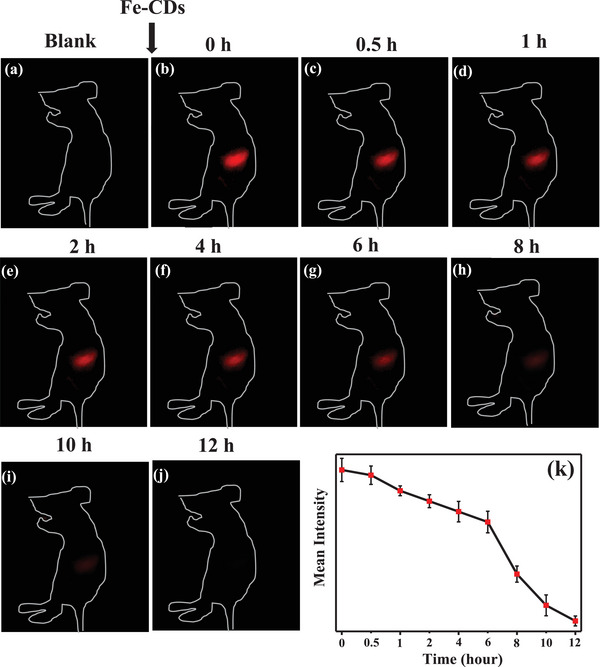
(a–j) NIR fluorescence imaging of mice orally administered with 100 µL Fe‐CDs (2 mg mL^−1^), and monitored over a period of 12 h; k) quantitation of mean fluorescence intensity.

To determine the maximum duration of in vivo fluorescence imaging using Fe‐CDs, we continuously monitored changes in fluorescence signal for up to 12 h in mice orally administered with Fe‐CDs (**Figure** **7**). Obvious fluorescence signals were observed in the first 6 h, which then began to fade and completely disappeared after 12 h. The Fe‐CDs were likely metabolized and cleared from the body 12 h after ingestion, which was reflective of their biocompatibility. This extended time of in vivo clearance of Fe‐CDs from the stomach was advantageous for long‐term bioimaging and biosensing, compared to the best available molecular fluorophores that could last for <2 h in the stomach.^[^ ^51^ ^]^

### 2.8 Histological Analysis

**Figure 8:**
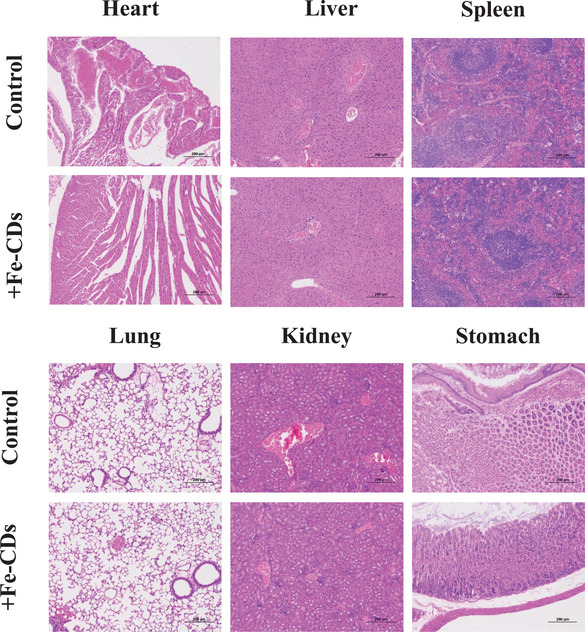
H&E images of tissue sections from different organs of mice fed with Fe‐CDs or PBS (control).

All mice were sacrificed to collect their internal organs, including the heart, liver, spleen, lung, kidney, and stomach. H&E staining was performed to evaluate any local or systemic inflammatory responses arising from Fe‐CDs ingestion (**Figure** **8**). No obvious inflammation reaction was observed in any of the organs from mice administered with Fe‐CDs, and similar tissue appearance was observed compared to control mice. These findings verify the good biocompatibility of Fe‐CDs.

## 3 Conclusion

We have developed new NIR‐II emssion Fe‐doped CDs with stable peak absorption/emission up to 830/1000 nm in acidic solution. The Fe‐CDs exhibited high QY and superior pH imaging performance compared to existing red emission CDs, with great potential to be applied for in vivo pH monitoring. It was demonstrated that the tissue penetration depth of the Fe‐CDs was ≈6 mm and pH imaging with reliable accuracy can be obtained when the tissue thickness is 4 mm or less. As an example of their biological applications, we demonstrated that the Fe‐CDs can be successfully applied for non‐invasive real‐time monitoring of gastric pH during food digestion in mice, and pH variation under the influence of antacid drugs (omeprazole). This study unlocks the potential of Fe‐CDs for simple, accurate, reliable, cost‐effective, and safe NIR‐II bioimaging and biosensing.

## 4 Experimental Section

### Materials

All reagents from commercial sources were used as received without further purification or modification unless otherwise stated. Dopamine hydrochloride (DA), o‐phenylenediamine (oPD), and FeCl_3_·6H_2_O were obtained from Sigma‐Aldrich. The amino acids glycine (Gly), valine (Val), lysine (Lys), serine (Ser), leucine (Leu), phenylalanine (Phe), glutamine (Gln), isoleucine (Ile), tyrosine (Tyr), tryptophan (Trp), threonine (Thr), methionine (Met), glutamate (Glu), alanine (Ala), aspartic acid (Asp), proline (Pro), glutamic acid (Glu), arginine (Arg), histidine (His), cysteine (Cys), asparagine (Asn), homocysteine (Hcys), vitamin C (Vc), and glutathione (GSH) were obtained from Shanghai Macklin Biochemical Co. Ltd. (Shanghai, China). Sodium chloride (NaCl), sodium hydrogen phosphate (Na_2_HPO_4_), and sodium phosphate monobasic (NaH_2_PO_4_) were obtained from Shanghai Lingfeng Chemical Reagent Co. Ltd. Omeprazole enteric capsules were purchased from Youcare Pharmaceutical Group Co. Ltd. (Beijing, China). Cell Counting Kit‐8 was purchased from Mycos Biotechnology Co., LTD (Shenzhen, China). Fluorescent dye IR‐26 was purchased from Qiyue Biological Technology Co. Ltd (Xian, China).

### Apparatus

Transmission electron microscopy (TEM) with FEI Tecnai G2 f20 s‐twin at 200 kV accelerating voltage was used to image the size of Fe‐CDs. X‐ray photoelectron spectroscopy (XPS) with Thermo Fisher Scientific K‐Alpha was used to characterize the elemental composition and bonding configuration of Fe‐CDs. Fourier transform infrared (FT‐IR) was performed using a VECTOR 22 spectrophotometer (Bruker, Germany). Zeta potential and hydrodynamic diameters were measured using Zetasizer Nano ZS (Malvern, Britain). Fe^3+^ concentration was determined using the Agilent 7700 ICP‐MS system (Agilent Technologies, Santa Clara, CA). Fluorescence spectra were recorded using a fluorescence spectrometer (F900, Edinburgh). Emission spectra were obtained at an excitation wavelength of 1000 nm. UV–Vis–NIR absorption spectra were recorded on Lamda750S PerkinElmer. The pH of the buffer solution was controlled by a digital pH meter (FE20, Mettler‐Toledo). An in‐house NIR‐II Fluorescence in Vivo Small‐Animal Imaging System was used, built using a 640 × 512 pixel 2D InGaAs/SWIR VGA standard camera (detection range 900–1700 nm, Photonic Science, UK) equipped with a 1000 nm long‐pass filter (Thorlabs FEL, Newton, NJ, USA). A NIR lens pair SWIR‐35 (Navitar, Rochester, NY, USA) was used to focus the image onto the photodetector. The excitation light was provided by an 808 nm diode laser (Changchun New Industries Optoelectronics Tech. Co., Ltd. China). The excitation power density at the imaging plane was 45 mW cm^−2^. The NIR‐II images were taken at a fixed exposure time of 200 ms, and Image J was used to process the images for any necessary corrections.

### Synthesis of CDs

DA (0.1 g) and oPD (0.1 g) were mixed in 15 mL ethyl alcohol, and the solution was briskly stirred until dissolved. The mixture was transferred into a poly(tetrafluoroethylene) Teflon‐lined autoclave (50 mL) and heated to 180 °C for 12 h. After cooling to room temperature, the product was obtained by centrifugation and washing twice with ethyl alcohol. The supernatant was transferred to a dialysis bag and subjected to dialysis for 2 days to remove unreacted materials. The solution in the dialysis bag was freeze‐dried to obtain CDs powder.

### Synthesis of Fe‐CDs

The same process was followed as above for synthesizing CDs, except that FeCl_3_·6H_2_O (0.05 g) was added to the mixture of DA (0.1 g) and oPD (0.1 g) in 15 mL ethyl alcohol.

### QY Measurement of Fe‐CDs

The QY of Fe‐CDs was determined using NIR‐II‐emissive IR‐26 dissolved in dichloroethane (QY = 0.5%) as reference. The QY of Fe‐CDs was estimated using the following equation:^[^ ^39^ ^]^

(1) $$\Phi_{s}=\Phi_{r}\left(F_{s}/F_{r}\right)\times \left(A_{r}/A_{s}\right)\times \left(n_{s}^{2}/n_{r}^{2}\right)$$

where *Φ* is QY, F is NIR‐II‐emitting intensity, A is the absorption value at 808 nm wavelength, and n represents the refractive index of solvent (water: 1.33; dichloroethane: 1.44). The subscripts s and r indicate the CDs sample and IR‐26 reference, respectively.

### pH Detection

Different pH values of 2.0, 3.0, 4.0, 5.0, 6.0, 7.0, 8.0, 9.0, and 10.0 were detected in PBS buffer solution (10 mm). In pH detection experiments, ethanol solution containing Fe‐CDs (1 µL, 5 mg mL^−1^) was added to the PBS buffer (500 µL). The solution was mixed using a vortex and incubated at room temperature. Fluorescence spectra were obtained with excitation at 830 nm and emission at 1100 nm.

### Cytotoxicity Assay

4T1, 293T cells, and Raw 264.7 cells were seeded at 10 × 104 cells per well in 96‐well plates and cultured overnight. To detect cytotoxicity, cells were incubated with CDs at different mass concentrations (0–0.2 mg mL^−1^) for 24 h. Cell viability was estimated using the standard Cell Counting Kit‐8 (CCK‐8) method. The mean and standard deviation were calculated from five wells tested in parallel.

### Animal Experiments

All animal experiments were performed using protocols approved by the Animal Care and Use Committee (Shenzhen Institutes of Advanced Technology, Chinese Academy of Sciences; Serial number: SIAT‐IACUC‐20210312‐YYS‐NMZX‐ZPF‐A1715‐01).

### NIR‐II Penetration Depth in Tissue

Ethanol solution containing Fe‐CDs (100 µL, 2 mg mL^−1^) was added to the PBS buffer (pH = 2), and the mixed solution was then added to a 24‐well plate. CDs (100 µL, 2 mg mL^−1^) were used as a control group. Pork tissue slices with different thicknesses (0 to 7 mm) were used to cover the culture plate before imaging using an 808 nm diode laser with a fluence rate of ≈200 mW cm^−2^. The emission signals were collected at 1000 nm with an exposure time of 50 ms. Ratio images were processed using Matlab software, and average ratios were taken from the same region of interest in different images.

### Quantitative pH Measurements in Gastric Fluid

Female BALA mice (8 weeks old, 26–28 g), purchased from Shanghai SLRC laboratory animal center were fasted for 12 h with free access to water before in vivo experiments. Mice were randomly selected from cages for all experiments. Mice were randomly divided into five groups (n = 3), and each group was orally administered with 100 µL Fe‐CDs (2 mg mL^−1^) in PBS of different pH (2, 3, 4, 5, 6 respectively), followed by imaging. The excitation source was provided by an 808 nm diode laser with a fluence rate of ≈200 mW cm^−2^, and the emission signals were collected at 1000 nm with an exposure time of 50 ms. Ratio images were processed using Matlab software, and average ratios were taken from the same region of interest in various images.

### In Vivo NIR Imaging

The same mice as above were used and subjected to the same acclimatization procedures. Mice were randomly selected from cages for all experiments. Mice were randomly divided into two groups (n = 3), both orally administered with 100 µL Fe‐CDs (2 mg mL^−1^), and fed after 10 min. Then, one group was orally administered 50 µL Omeprazole (OMG) water solution (20 mg mL^−1^), and the other group was orally administered 50 µL water. NIR imaging of mice was conducted at different times following feeding. For non‐invasive imaging, all mice were anesthetized using a rodent ventilator with 2 L min^−1^ air mixed with 4% isoflurane, and imaging was performed at the left lateral aspect of the mouse abdomen. For invasive imaging, mice were sacrificed and dissected along the upper part of the abdomen. The stomach was exposed outside of the abdomen for imaging. For gastric fluid imaging, the excised stomachs were cut open along the greater curvature, excess gastric content was removed, and imaging was performed on the inner wall of the stomach. The excitation source was provided by an 808 nm diode laser with a fluence rate of ≈200 mW cm^−2^, and the emission signals were collected at 1000 nm with an exposure time of 50 ms. Ratio images were processed using Matlab software, and average ratios were taken from the same region of interest in various images.

### Histological Analysis

Explant stomach tissues were fixed in 4% neutral buffered formalin for 2 days and then embedded in paraffin after dehydration through an ethanol series. Sections (5 µm) were cut and dewaxed in xylene, dehydrated using an ascending ethanol gradient, and washed in PBS for 5 min. The sections were stained with hematoxylin and eosin (H&E). Neutral gum was used as the sealing piece for further morphological observation. All sections were imaged using a light microscope (Zeiss, Germany).

## Conflict of Interest

The authors declare no conflict of interest.

## Supporting information

Supporting Information

## Data Availability

The data that support the findings of this study are available from the corresponding author upon reasonable request.;

## References

[1] R. Wang , L. Zhou , W. Wang , X. Li , F. Zhang , Nat. Commun. 2017, 8, 14702. 28281530 10.1038/ncomms14702 PMC5353702

[2] I. M. Modlin , K. D. Lye , M. Kidd , Cancer 2003, 97, 934. 12569593 10.1002/cncr.11105

[3] P. R. Wood , P. G. P. Lawler , Br. J. Anaesth. 1996, 76, 563. 8652331 10.1093/bja/76.4.563

[4] S. Zhang , A. M. Bellinger , D. L. Glettig , R. Barman , Y. A. L. Lee , J. Zhu , C. Cleveland , V. A. Montgomery , L. Gu , L. D. Nash , D. J. Maitland , R. Langer , G. Traverso , Nat. Mater. 2015, 14, 1065. 26213897 10.1038/nmat4355 PMC4772966

[5] Y. Ma , Y. Liu , Z. Jiang , H. Lv , J. Wang , T. Wang , X. Zhang , Y. Hu , H. Lin , W. Lin , Sens. Actuators, B 2021, 349, 130747.

[6] G. Ma , X. Gao , C. Jiang , S. Xing , C. Wei , P. Huang , J. Lin , Anal. Chem. 2019, 91, 13570. 31610654 10.1021/acs.analchem.9b02701

[7] A. L. Antaris , H. Chen , S. Diao , Z. Ma , Z. Zhang , S. Zhu , J. Wang , A. X. Lozano , Q. Fan , L. Chew , M. Zhu , K. Cheng , X. Hong , H. Dai , Z. Cheng , Nat. Commun. 2017, 8, 15269. 28524850 10.1038/ncomms15269 PMC5454457

[8] C. Li , G. Chen , Y. Zhang , F. Wu , Q. Wang , J. Am. Chem. Soc. 2020, 142, 14789. 32786771 10.1021/jacs.0c07022

[9] P. Wang , Y. Fan , L. Lu , L. Liu , L. Fan , M. Zhao , Y. Xie , C. Xu , F. Zhang , Nat. Commun. 2018, 9, 2898. 30042434 10.1038/s41467-018-05113-8 PMC6057964

[10] Y. Tang , Y. Li , X. Hu , H. Zhao , Y. Ji , L. Chen , W. Hu , W. Zhang , X. Li , X. Lu , W. Huang , Q. Fan , Adv. Mater. 2018, 30, 1801140. 10.1002/adma.201801140 29920793

[11] Q. Yang , Z. Ma , H. Wang , B. Zhou , S. Zhu , Y. Zhong , J. Wang , H. Wan , A. Antaris , R. Ma , X. Zhang , J. Yang , X. Zhang , H. Sun , W. Liu , Y. Liang , H. Dai , Adv. Mater. 2017, 29, 1605497. 10.1002/adma.201605497 28117499

[12] W. Liu , L. Miao , X. Li , Z. Xu , Coord. Chem. Rev. 2021, 429, 213646.

[13] L. Yin , H. Sun , H. Zhang , L. He , L. Qiu , J. Lin , H. Xia , Y. Zhang , S. Ji , H. Shi , M. Gao , J. Am. Chem. Soc. 2019, 141, 3265. 30689382 10.1021/jacs.8b13628

[14] S. Wang , Y. Fan , D. Li , C. Sun , Z. Lei , L. Lu , T. Wang , F. Zhang , Nat. Commun. 2019, 10, 1058. 30837470 10.1038/s41467-019-09043-x PMC6401027

[15] B. Guo , J. Chen , N. Chen , E. Middha , S. Xu , Y. Pan , M. Wu , K. Li , C. Liu , B. Liu , Adv. Mater. 2019, 31, 1902504. 10.1002/adma.201808355 31063244

[16] G. Hong , Y. Zou , A. L. Antaris , S. Diao , D. Wu , K. Cheng , X. Zhang , C. Chen , B. Liu , Y. He , J. Z. Wu , J. Yuan , B. Zhang , Z. Tao , C. Fukunaga , H. Dai , Nat. Commun. 2014, 5, 4206. 24947309 10.1038/ncomms5206

[17] G. Hong , J. C. Lee , J. T. Robinson , U. Raaz , L. Xie , N. F. Huang , J. P. Cooke , H. Dai , Nat. Med. 2012, 18, 1841. 23160236 10.1038/nm.2995 PMC3595196

[18] G. Hong , S. Diao , J. Chang , A. L. Antaris , C. Chen , B. Zhang , S. Zhao , D. N. Atochin , P. L. Huang , K. I. Andreasson , C. J. Kuo , H. Dai , Nat. Photonics 2014, 8, 723. 27642366 10.1038/nphoton.2014.166 PMC5026222

[19] Y. Fan , P. Wang , Y. Lu , R. Wang , L. Zhou , X. Zheng , X. Li , J. A. Piper , F. Zhang , Nat. Nanotechnol. 2018, 13, 941. 30082923 10.1038/s41565-018-0221-0

[20] R. Wang , X. Li , L. Zhou , F. Zhang , Angew. Chem. 2014, 126, 12282. 10.1002/anie.201407420 25196421

[21] C. Li , Y. Zhang , M. Wang , Y. Zhang , G. Chen , L. Li , D. Wu , Q. Wang , Biomaterials 2014, 35, 393. 24135267 10.1016/j.biomaterials.2013.10.010

[22] Z. Sheng , B. Guo , D. Hu , S. Xu , W. Wu , W. H. Liew , K. Yao , J. Jiang , C. Liu , H. Zheng , B. Liu , Adv. Mater. 2018, 30, 1800766. 10.1002/adma.201800766 29806179

[23] S. Yu , Y. Zhou , Y. Sun , S. Wu , T. Xu , Y. C. Chang , S. Bi , L. P. Jiang , J. J. Zhu , Angew. Chem., Int. Ed. 2021, 60, 5948. 10.1002/anie.202012801 33289255

[24] Y. Chen , L. Xue , Q. Zhu , Y. Feng , M. Wu , Front. Chem. 2021, 9, 750404. 34733821 10.3389/fchem.2021.750404 PMC8558517

[25] F. Ding , Y. Fan , Y. Sun , F. Zhang , Adv. Healthcare Mater. 2019, 8, 1900260. 10.1002/adhm.201900260 30983165

[26] H. Ding , X. X. Zhou , J. S. Wei , X. B. Li , B. T. Qin , X. B. Chen , H. M. Xiong , Carbon 2020, 167, 322.

[27] S. Li , W. Su , H. Wu , T. Yuan , C. Yuan , J. Liu , G. Deng , X. Gao , Z. Chen , Y. Bao , F. Yuan , S. Zhou , H. Tan , Y. Li , X. Li , L. Fan , J. Zhu , A. T. Chen , F. Liu , Y. Zhou , M. Li , X. Zhai , J. Zhou , Nat. Biomed. Eng. 2020, 4, 704. 32231314 10.1038/s41551-020-0540-y PMC7197249

[28] N. Gong , X. Ma , X. Ye , Q. Zhou , X. Chen , X. Tan , S. Yao , S. Huo , T. Zhang , S. Chen , X. Teng , X. Hu , J. Yu , Y. Gan , H. Jiang , J. Li , X. J. Liang , Nat. Nanotechnol. 2019, 14, 379. 30778211 10.1038/s41565-019-0373-6

[29] X. Guo , C. F. Wang , Z. Y. Yu , L. Chen , S. Chen , Chem. Commun. 2012, 48, 2692. 10.1039/c2cc17769b 22306963

[30] J. Shen , Y. Zhu , X. Yang , C. Li , Chem. Commun. 2012, 48, 3686. 10.1039/c2cc00110a 22410424

[31] C. Liu , P. Zhang , X. Zhai , F. Tian , W. Li , J. Yang , Y. Liu , H. Wang , W. Wang , W. Liu , Biomaterials 2012, 33, 3604. 22341214 10.1016/j.biomaterials.2012.01.052

[32] L. Cao , X. Wang , M. J. Meziani , F. Lu , H. Wang , P. G. Luo , Y. Lin , B. A. Harruff , L. M. Veca , D. Murray , S. Y. Xie , Y. P. Sun , J. Am. Chem. Soc. 2007, 129, 11318. 17722926 10.1021/ja073527l PMC2691414

[33] J. Zhang , S. H. Yu , Mater. Today 2016, 19, 382.

[34] S. Zhu , Q. Meng , L. Wang , J. Zhang , Y. Song , H. Jin , K. Zhang , H. Sun , H. Wang , B. Yang , Angew. Chem. 2013, 125, 4045. 10.1002/anie.201300519 23450679

[35] K. Hola , Y. Zhang , Y. Wang , E. P. Giannelis , R. Zboril , A. L. Rogach , Nano Today 2014, 9, 590.

[36] P. Huang , J. Lin , X. Wang , Z. Wang , C. Zhang , M. He , K. Wang , F. Chen , Z. Li , G. Shen , D. Cui , X. Chen , Adv. Mater. 2012, 24, 5104. 22718562 10.1002/adma.201200650 PMC3657566

[37] H. Li , X. He , Z. Kang , H. Huang , Y. Liu , J. Liu , S. Lian , C. H. A. Tsang , X. Yang , S. T. Lee , Angew. Chem., Int. Ed. 2010, 49, 4430. 10.1002/anie.200906154 20461744

[38] H. Li , R. Liu , S. Lian , Y. Liu , H. Huang , Z. Kang , Nanoscale 2013, 5, 3289. 23467384 10.1039/c3nr00092c

[39] Y. Li , G. Bai , S. Zeng , J. Hao , ACS Appl. Mater. Interfaces 2019, 11, 4737. 30644718 10.1021/acsami.8b14877

[40] T. Han , Y. Wang , S. Ma , M. Li , N. Zhu , S. Tao , J. Xu , B. Sun , Y. Jia , Y. Zhang , S. Zhu , B. Yang , Adv. Sci. 2022, 9, 2203474. 10.1002/advs.202203474 PMC9596834 36047633

[41] X. Miao , X. Yan , D. Qu , D. Li , F. F. Tao , Z. Sun , ACS Appl. Mater. Interfaces 2017, 9, 18549. 28508626 10.1021/acsami.7b04514

[42] X. Miao , D. Qu , D. Yang , B. Nie , Y. Zhao , H. Fan , Z. Sun , Adv. Mater. 2018, 30, 1704740. 10.1002/adma.201704740 29178388

[43] H. Yıldırım , N. Bayrak , M. Yıldız , F. N. Yılmaz , E. Mataracı‐Kara , D. Shilkar , V. Jayaprakash , A. F. Tuyun , Molecules 2923, 2022, 27. 35566274 10.3390/molecules27092923 PMC9104734

[44] M. M. Abdelghafour , Á. Orbán , Á. Deák , Ł. Lamch , É. Frank , R. Nagy , A. Ádám , P. Sipos , E. Farkas , F. Bari , L. Janovák , Polymers 2021, 13, 2725. 34451264 10.3390/polym13162725 PMC8398594

[45] H. Namduri , S. Nasrazadani , Corros. Sci. 2008, 50, 2493.

[46] S. Lu , L. Sui , J. Liu , S. Zhu , A. Chen , M. Jin , B. Yang , Adv. Mater. 2017, 29, 1603443. 10.1002/adma.201603443 28195369

[47] Q. Zhang , R. Wang , B. Feng , X. Zhong , K. (Ken) Ostrikov , Nat. Commun. 2021, 12, 6856. 34824216 10.1038/s41467-021-27071-4 PMC8616937

[48] X. Ye , Y. Xiang , Q. Wang , Z. Li , Z. Liu , Small 2019, 15, 1901673. 10.1002/smll.201901673 31157517

[49] A. Moonen , J. Aguilera‐Lizarraga , R. Bisschops , P. Moonen , J. Tack , G. E. Boeckxstaens , Neurogastroenterol. Motil. 2019, 31, 1901673. 10.1111/nmo.13694 31449342

[50] Y. Ning , S. Cheng , J. X. Wang , Y. W. Liu , W. Feng , F. Li , J. L. Zhang , Chem. Sci. 2019, 10, 4227. 31057751 10.1039/c9sc00220k PMC6471977

[51] E. L. McConnell , A. W. Basit , S. Murdan , J. Pharm. Pharmacol. 2010, 60, 63. 10.1211/jpp.60.1.0008 18088506

