# Supplementary material for: Fe‐Doped Carbon Dots as NIR‐II Fluorescence Probe for In Vivo Gastric Imaging and pH Detection
Source: Adv Sci (Weinh). 2023 Jan 3;10(7):2206271. doi: 10.1002/advs.202206271 (PMC9982550; doi:10.1002/advs.202206271)
Supplement: Supplementary file 1 — Supporting Information [file ADVS-10-2206271-s001.pdf]

ADVANCED SCIENCE
Open Access

Supporting Information

for *Adv. Sci.*, DOI 10.1002/advs.202206271

Fe-Doped Carbon Dots as NIR-II Fluorescence Probe for In Vivo Gastric Imaging and pH Detection

Qiaoqiao Ci, Yuanyuan Wang, Ben Wu, Emerson Coy, Jiao jiao Li, Daoyong Jiang, Pengfei Zhang and Guocheng Wang**

Supporting Information

Fe-doped carbon dots as NIR-II fluorescence probe for *in vivo* gastric imaging and pH detection

Qiaoqiao Ci[a#], Yuanyuan Wang[b#], Ben Wu[a#], Emerson Coy[c], Jiao jiao Li[d], Daoyong Jiang[b] Pengfei Zhang[b], Guocheng Wang[a*]*

Table S1. ICP results of different sample groups.

Sample	Concentration Fe^{3+} (PPM)	RSD
Blank	0	/
DA+OPD	0	/
DA+OPD+Fe^{3+}	80.2	1.243647

Table S2. Functional group percentages of Fe-CDs and CDs measured by XPS.

	CDs		Fe-CDs	
**Name**	**Functional group**	**Area (%)**	**Functional group**	**Area (%)**
C1s	C-C/C=C	66.83	C-C/C=C	10.52
	C-N/C-O	32.83	C-N/C-O	79.87
	C=O/C=N	0.64	C=O/C=N	9.61
N1s	pyridinic N	56.55	pyridinic N	31.42

	pyrrolic N	5.74	pyrrolic N	2.14
	**graphitic N**	**37.71**	**graphitic N**	**66.44**
**O1s**	**C=O**	**32.23**	**C=O**	**61.85**
	C-O	67.77	C-O	38.15

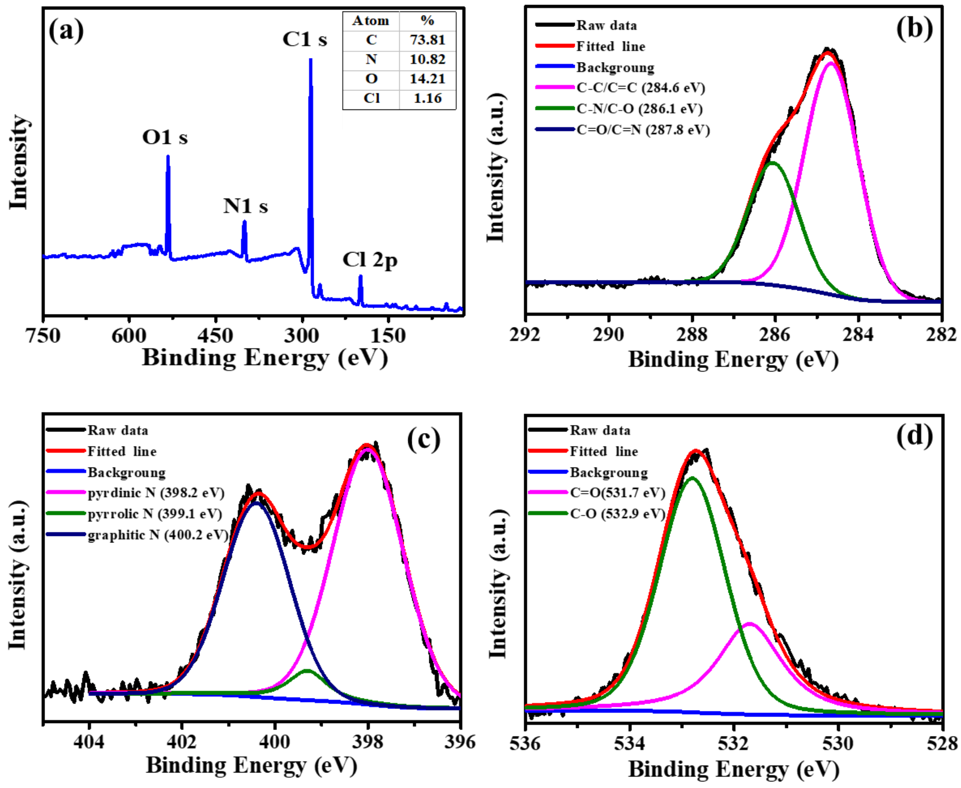

Figure S1. Characterization of CDs synthesized under the same experimental conditions as Fe-CDs but without adding Fe^{3+}, by (a) XPS spectrum, and (b) C 1s, (c) N 1s, (d) O 1s spectra.

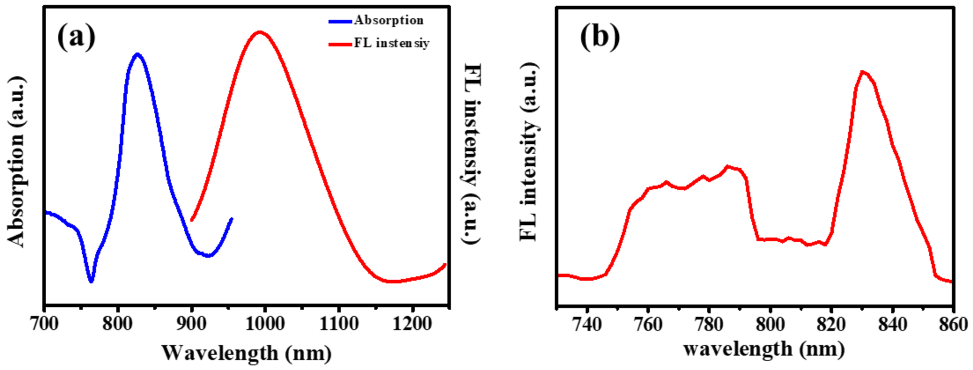

Figure S2. (a) UV absorption and fluorescence emission spectra of Fe-CDs in PBS buffer (10 mM, pH 2.0). (b) Fluorescence excitation spectrum of Fe-CDs in PBS buffer (λem=1000 nm) .

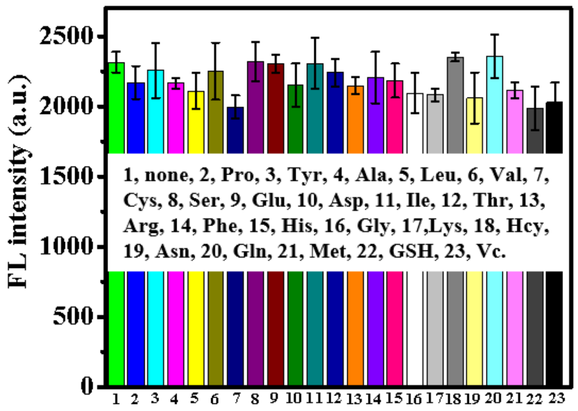

Figure S3. Fluorescence intensity in solutions containing various amino acids and biothiols (200 μM) in PBS buffer (10 mM, pH 2.0).

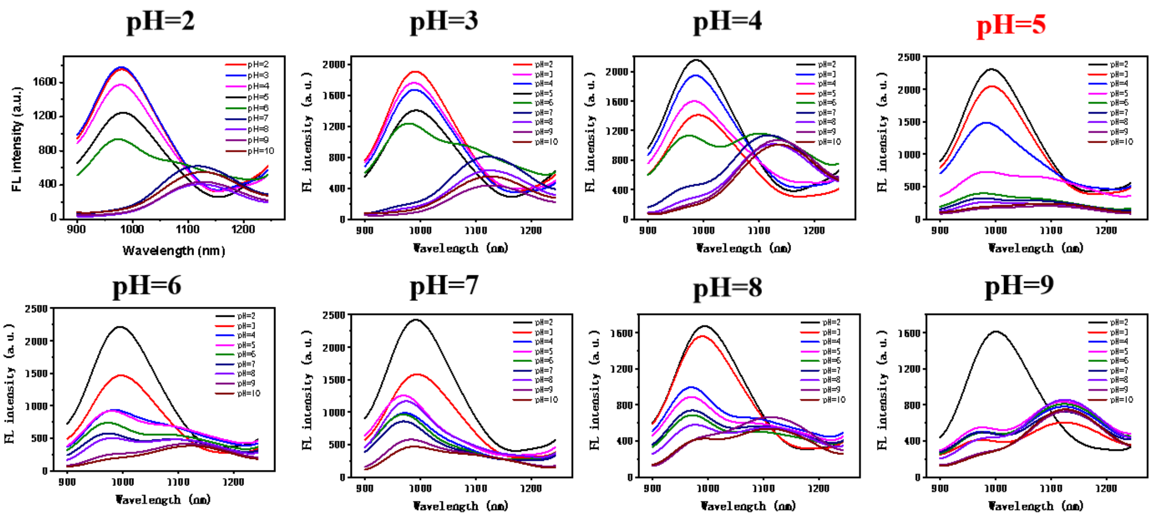

Figure S4. Fluorescence emission spectra of Fe-CDs synthesized under reaction conditions with different pH of 2.0 to 9.0 (λex = 830 nm).

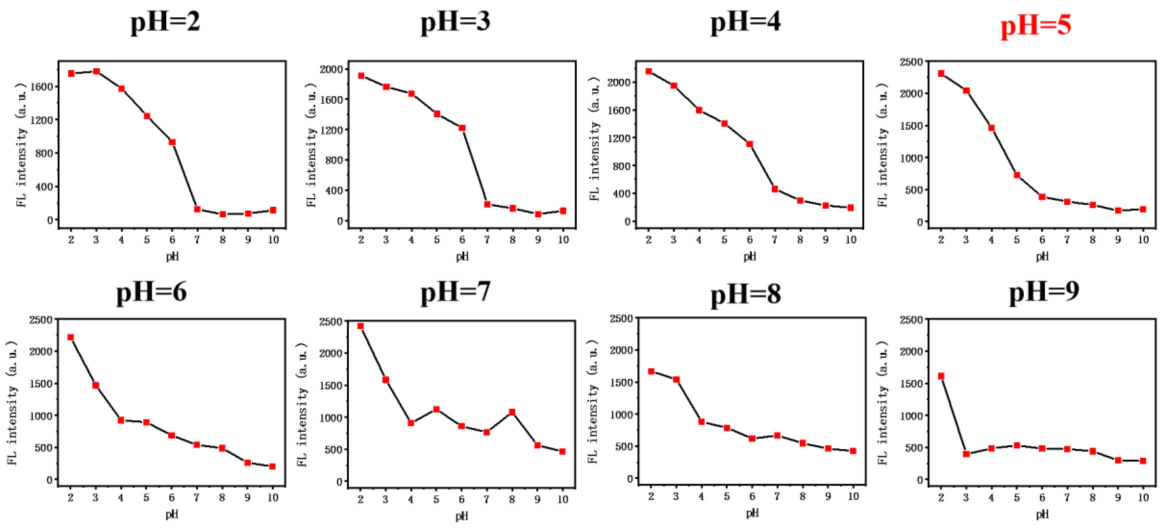

Figure S5. Relationship between the fluorescence intensity and the pH value of Fe-CDs synthesized under different pH reaction solutions (λem = 1000 nm).

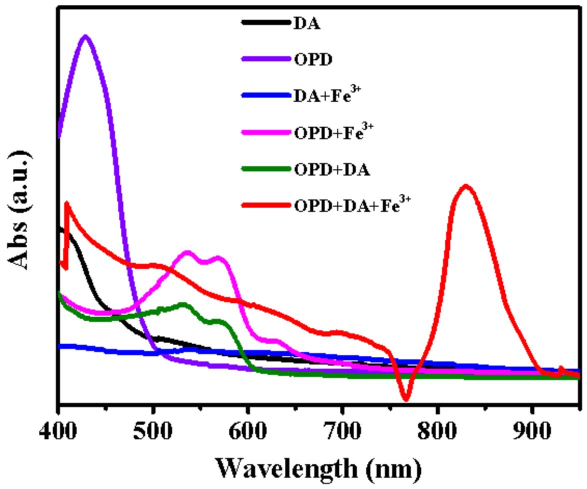

Figure S6. UV absorption of different combinations of reactants for synthesizing CDs in PBS buffer (10 mM, pH 2.0).

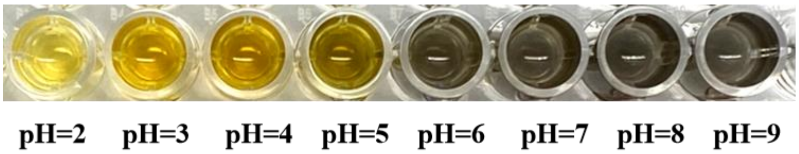

Figure S7. Colorimetric images of Fe-CDs at different pH values.

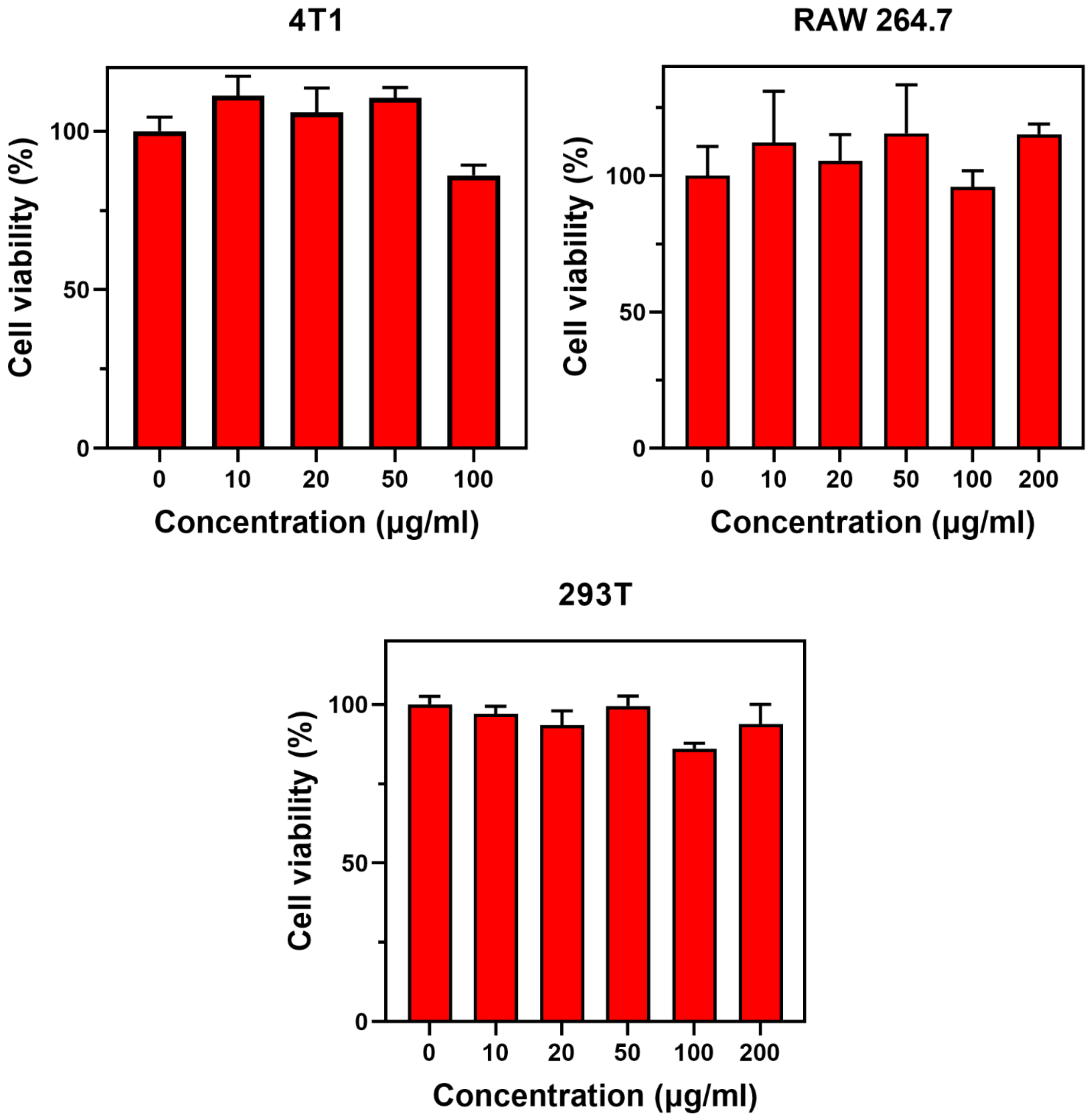

Figure S8. Cytotoxicity evaluation of Fe-CDs.

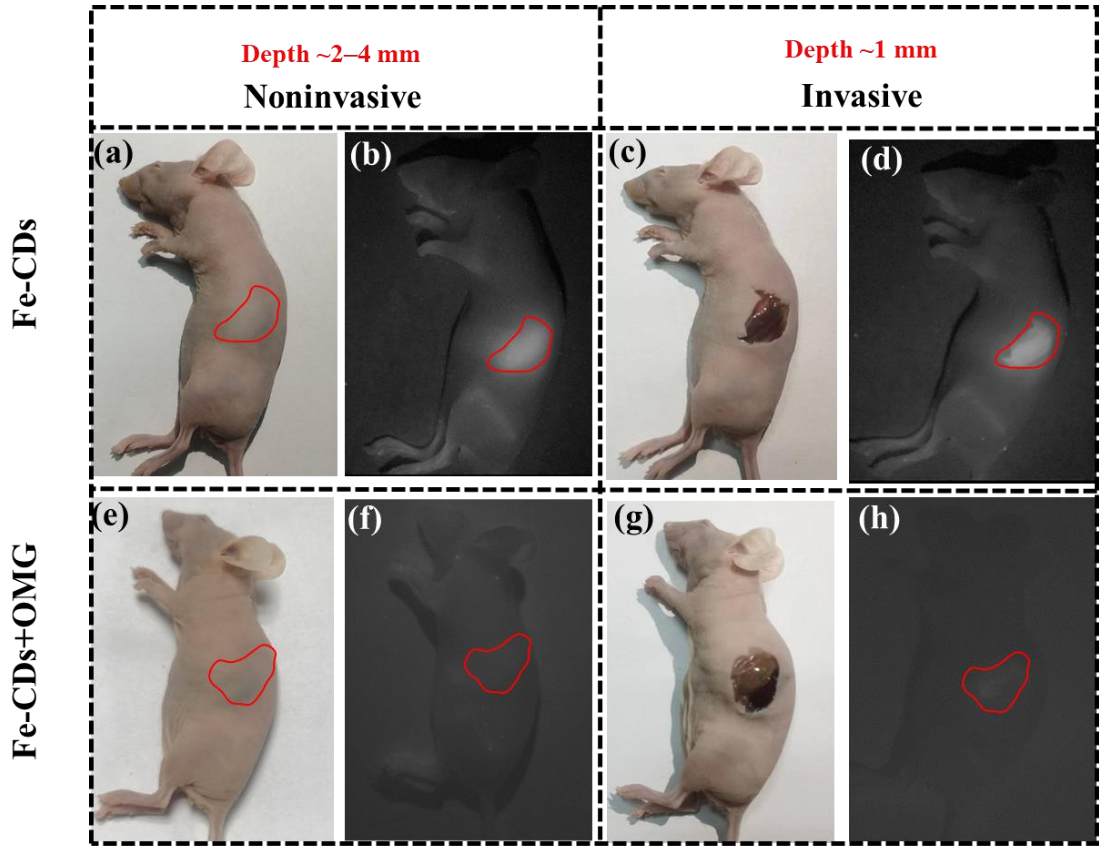

Figure S9. Mice were imaged after being orally administered with 100 μL Fe-CDs (2 mg/mL), with or without 50 μL OMG (20 mg/mL). (**a, b, e, f**) Digital photographs and NIR fluorescence imaging for non-invasive imaging at 2–4 mm tissue depth; (**c, d, g, h**) invasive imaging of gastric fluid covered by ~1 mm thick gastric wall.

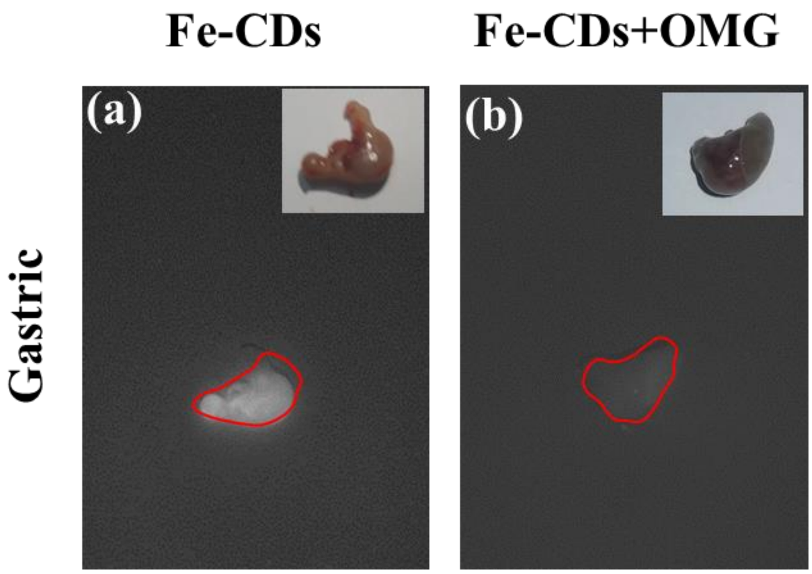

Figure S10. Direct imaging of exposed gastric fluid from dissected stomachs of mice orally administered with 100 μL Fe-CDs (2 mg/mL), with or without 50 μL OMG (20 mg/mL).

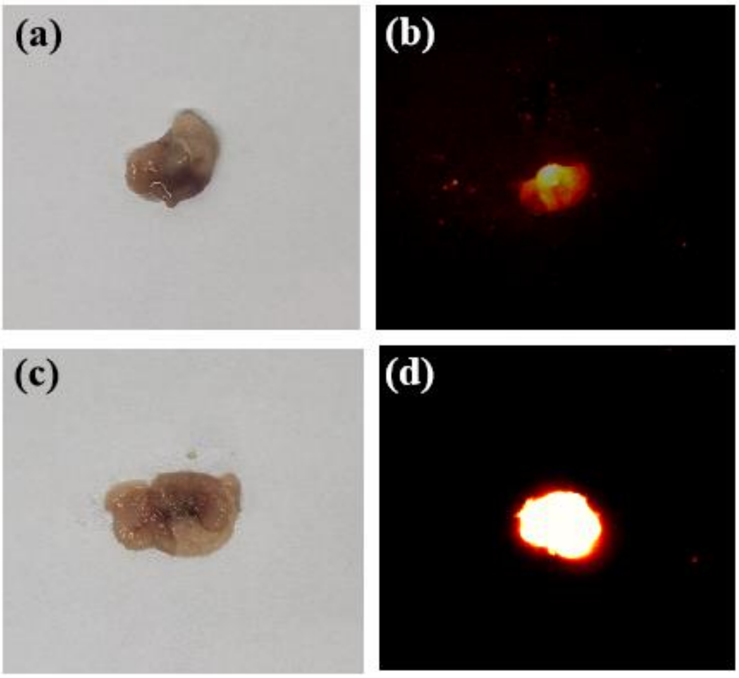

Figure S11. Digital photographs and NIR fluorescence imaging of (**a, b**) dissected mouse stomach, (**c, d**) inner wall of mouse stomach after removing gastric content.

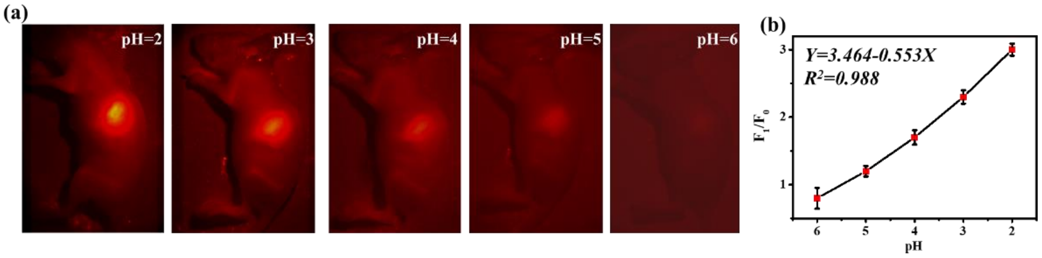

Figure S12. (a) NIR imaging of mice orally administered with 100 μL Fe-CDs (2 mg/mL) in PBS of different pH (2 to 6). (b) Linear relationship of fluorescence intensity ratio of Fe-CDs versus pH value in the pH range 2 to 6.
